# Supplementary material for: Circa-SCOPE: high-throughput live single-cell imaging method for analysis of circadian clock resetting
Source: Nat Commun. 2021 Oct 8;12:5903. doi: 10.1038/s41467-021-26210-1 (PMC8501123; doi:10.1038/s41467-021-26210-1)
Supplement: Supplementary file 1 — Supplementary Information [file 41467_2021_26210_MOESM1_ESM.pdf]

Supplementary Information:

Circa-SCOPE: high-throughput live single-cell imaging method for analysis of circadian clock resetting

Gal Manella[1], Dan Aizik[1], Rona Aviram[1], Marina Golik[1] and Gad Asher[1]*

[1]Department of Biomolecular Sciences, Weizmann Institute of Science, 7610001, Rehovot, Israel.

* Lead Contact and Corresponding Author: gad.asher@weizmann.ac.il

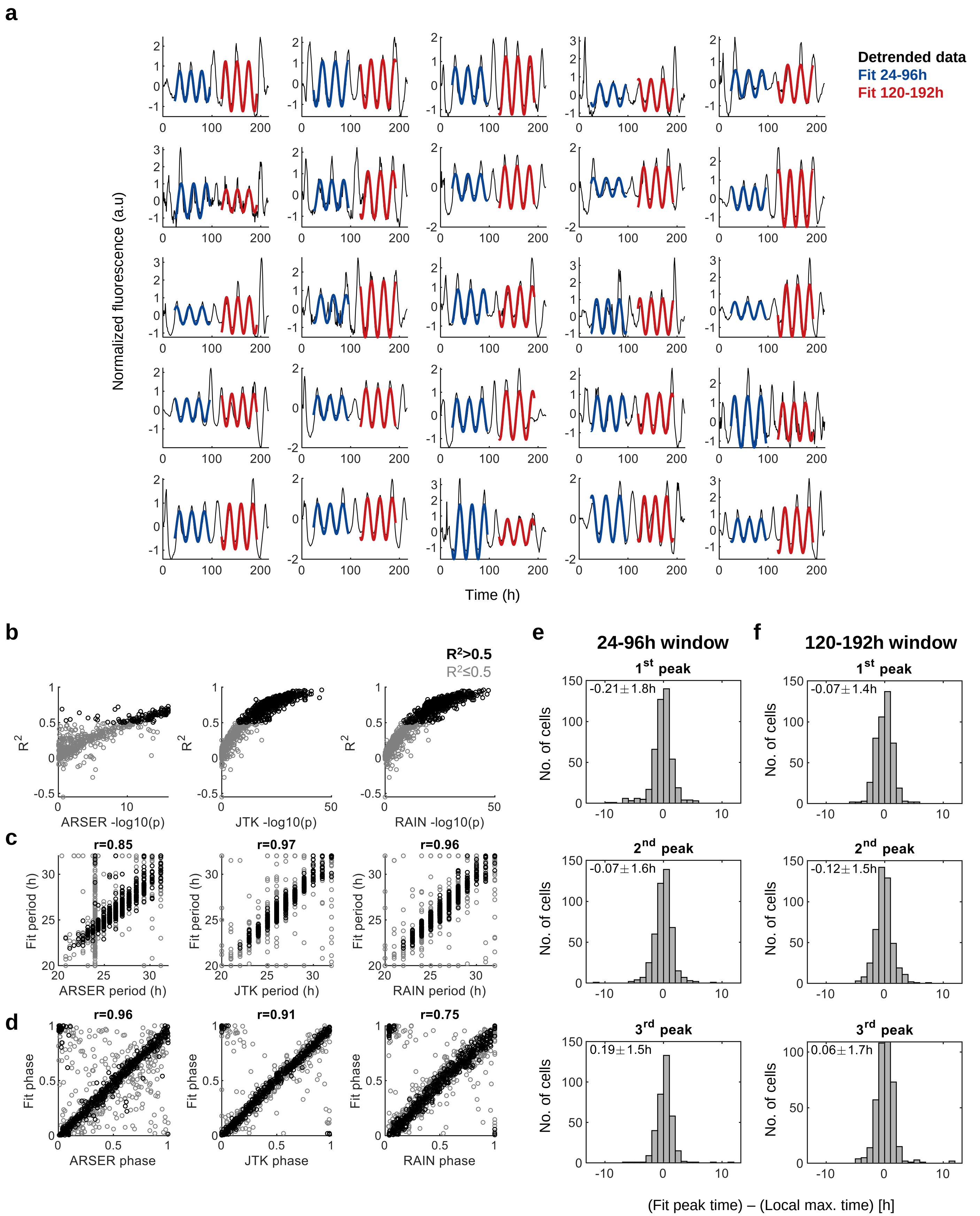
a
Detrended data
Fit 24-96h
Fit 120-192h
Normalized fluorescence (a.u)
Time (h)
b
R²>0.5
R²≤0.5
ARSER -log10(p)
JTK -log10(p)
RAIN -log10(p)
c
r=0.85
r=0.97
r=0.96
Fit period (h)
ARSER period (h)
JTK period (h)
RAIN period (h)
d
r=0.96
r=0.91
r=0.75
Fit phase
ARSER phase
JTK phase
RAIN phase
e
24-96h window
f
120-192h window
1st peak
-0.21±1.8h
-0.07±1.4h
2nd peak
-0.07±1.6h
-0.12±1.5h
3rd peak
0.19±1.5h
0.06±1.7h
No. of cells
(Fit peak time) – (Local max. time) [h]

Supp. Fig. 1

Supplementary Figure 1. Rhythmicity analysis evaluation

(a) Representative detrended traces with their cosine-fits in two windows: 24-96h (blue), or 120-192h (red). (R^2>0.5 for all fits).

(b-d) Comparison of rhythmicity analysis using the cosine-model fitting with different independent methods; ARSER, JTK, and RAIN. The data in the time window between 24-96h in Fig. 1 was either fitted with a cosine or analyzed with the different indicated methods.

(b) Comparison of the R^2 of the cosine fit with the p-value obtained in each of the methods. Each point represents a single cell (n=569 with R^2>0.5, out of 1011 cells).

(c) Comparison of the period estimates from the cosine fit with the period estimates from the different methods (r- Pearson's correlation coefficient, on the R^2>0.5 data).

(d) Comparison of the phase estimates from the cosine fit and the phase estimates from the different methods (phases are given relative to the period length, r- circular correlation coefficient, on the R^2>0.5 data).

(e-f) Comparison of the peak times as extracted from the cosine fit with the local maxima times in their vicinity (i.e., 0 indicates perfect resemblance), either in the window 24-96h **(e)** or 120-192h **(f)** (Mean±SD is indicated for each histogram).

Source data are provided as Source Data file.

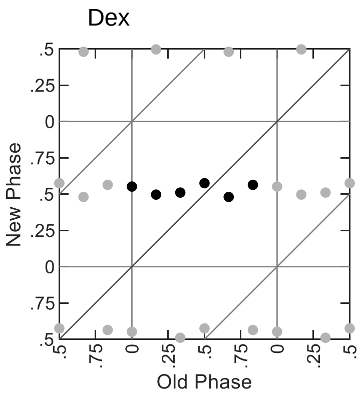

(NIH3T3 Bmal1-Luc-1)

Supp. Fig. 2

Supplementary Figure 2. Dexamethasone PTC reconstructed using the conventional approach

PTC reconstructed from bioluminescence data of NIH-3T3 Bmal1-luc-1 cell cultures treated with 100nM dexamethasone in 6 different time points along the circadian cycle. Depicted are mean old and new phases of 3 replicates per time point.

Source data are provided as Source Data file.

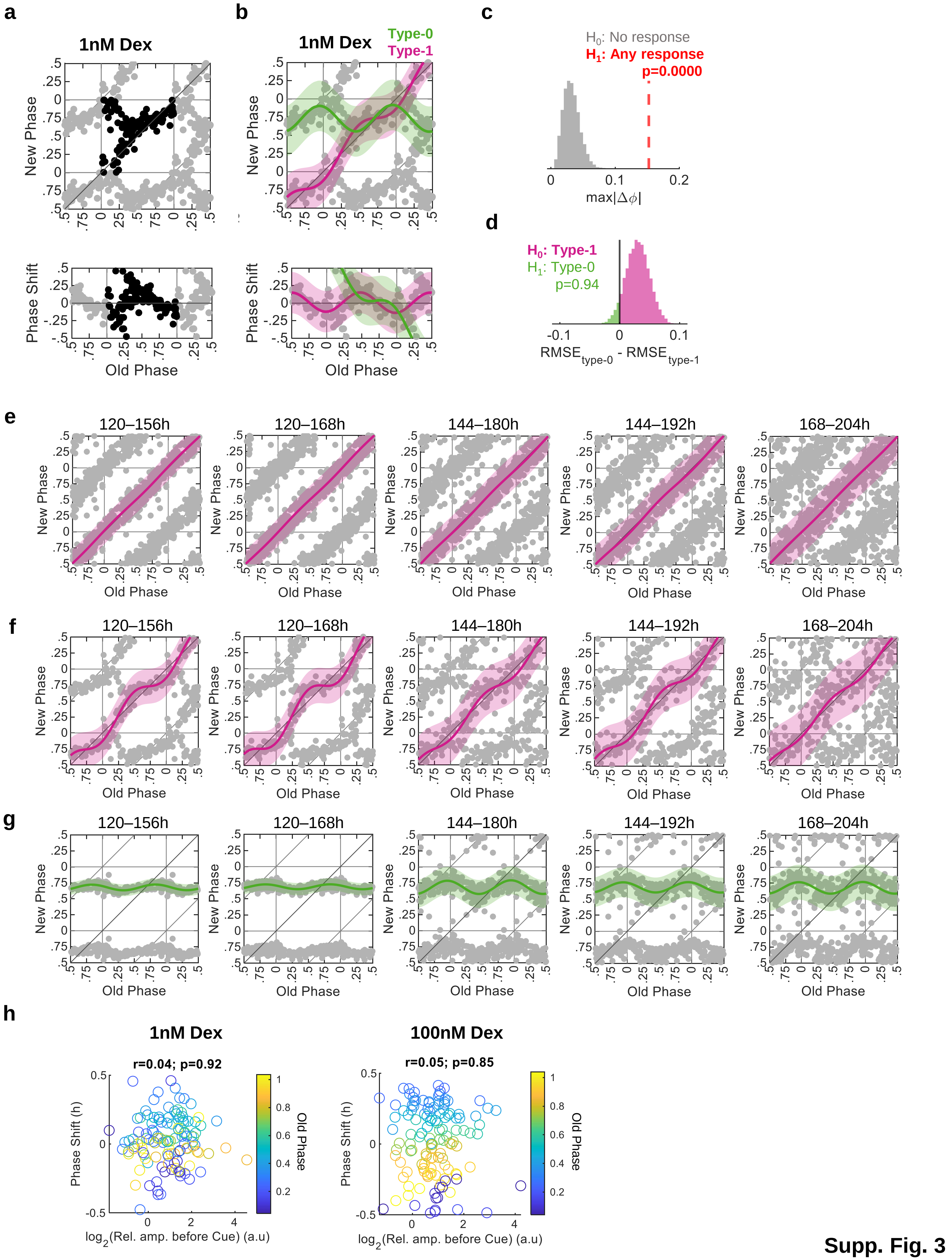
a
1nM Dex
New Phase
Phase Shift
Old Phase
b
1nM Dex
Type-0
Type-1
New Phase
Phase Shift
Old Phase
c
H0: No response
H1: Any response
p=0.0000
max|Δϕ|
d
H0: Type-1
H1: Type-0
p=0.94
RMSEtype-0 - RMSEtype-1
e
120–156h
120–168h
144–180h
144–192h
168–204h
f
120–156h
120–168h
144–180h
144–192h
168–204h
g
120–156h
120–168h
144–180h
144–192h
168–204h
h
1nM Dex
r=0.04; p=0.92
100nM Dex
r=0.05; p=0.85
Phase Shift (h)
Old Phase
log2(Rel. amp. before Cue) (a.u)

Supp. Fig. 3

Supplementary Figure 3. PTC reconstruction using Circa-SCOPE

(a-c) The same analysis as in Fig. 2c-g with lower concentration of Dexamethasone (Dex) (1nM).

(a) PTC (upper panel) and PRC (lower panel) representations of culture treated with 1nM Dex, (n=121 cells).

(b) Same data as in (a), fitted with two Fourier-based models, one for type-1 resetting (pink) and another for type-0 resetting (green). Fits are presented ±95% confidence intervals.

(c) Bootstrapping test for the response significance. The maximal absolute phase shift of the type-1 fit of untreated control was resampled 10,000 times and compared to the measured value of dexamethasone-treated fit.

(d) Bootstrapping test for the selection between type-1 and type-0 models. The dexamethasone-treated data was resampled 10,000 times, each time fitted with both models, and RMSE (Root Mean Square Error) was calculated for both. The model with the lower RMSE is selected, hence the p-value for type-0 is the probability of having ($RMSE_{type-0} - RMSE_{type-1}$) >0.

(e-g) Comparison of the PTCs reconstructed based on fitting in different after-windows, for untreated **(e)** 1nM Dex-treated **(f)** or 100nM Dex-treated **(g)**. Based on the same data as in Fig. 2 and in **(a-c).** data was fitted with type-1 (pink) or type-0 (green) Fourier-based models. Fits are presented ±95% confidence intervals.

(h) Dex-induced phase shift plotted against the instantaneous amplitude before the cue. The amplitudes were extracted as the difference between the local maxima and minima of the raw data, and normalized to the mean amplitude for the purpose of plotting. (r: the correlation coefficient between one circular and one linear variables, p: its significance level based on χ^2 test).

Source data are provided as Source Data file and in Supplementary Dataset 1.

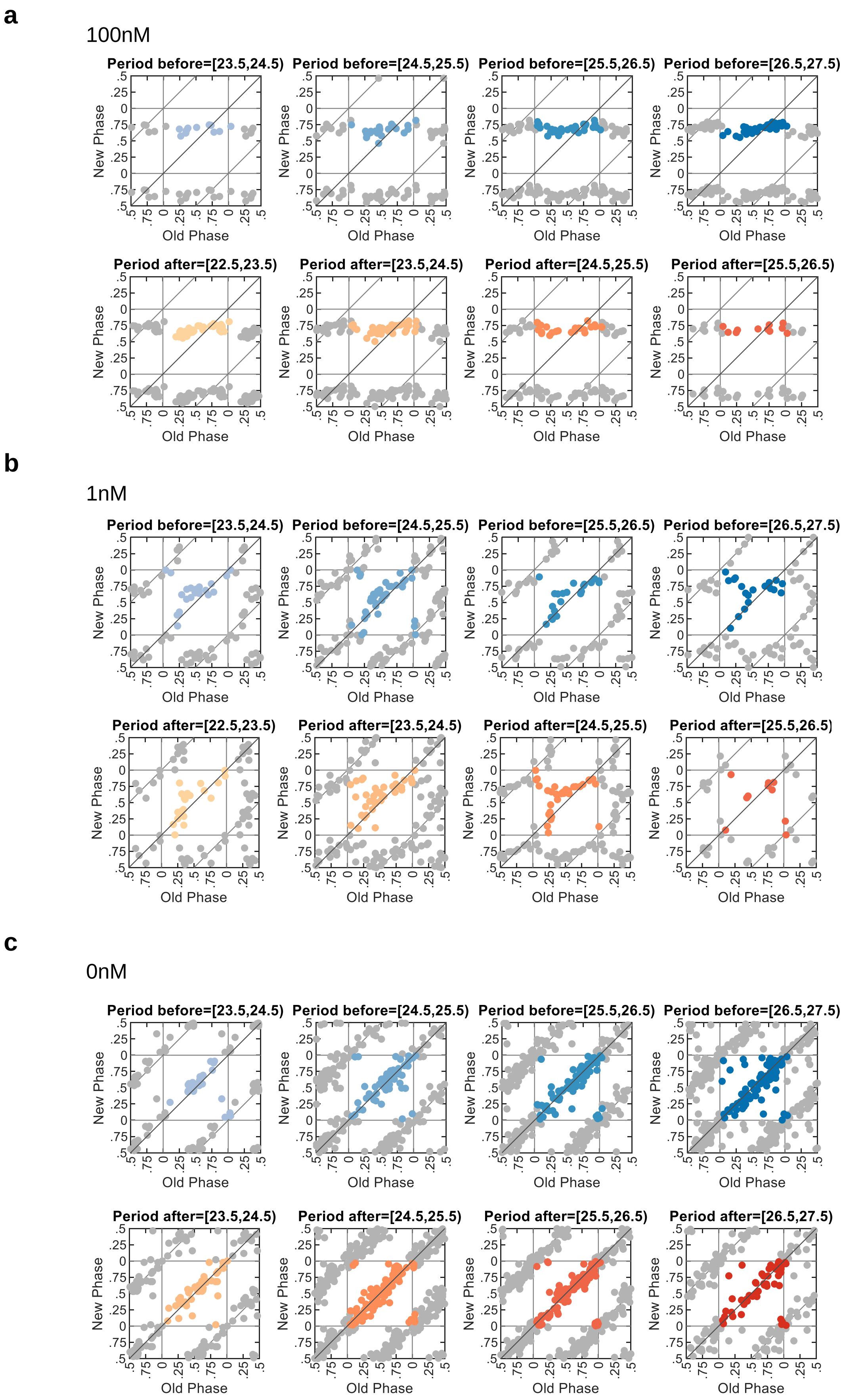
a
100nM
Period before=[23.5,24.5)
Period before=[24.5,25.5)
Period before=[25.5,26.5)
Period before=[26.5,27.5)
Period after=[22.5,23.5)
Period after=[23.5,24.5)
Period after=[24.5,25.5)
Period after=[25.5,26.5)
b
1nM
Period before=[23.5,24.5)
Period before=[24.5,25.5)
Period before=[25.5,26.5)
Period before=[26.5,27.5)
Period after=[22.5,23.5)
Period after=[23.5,24.5)
Period after=[24.5,25.5)
Period after=[25.5,26.5)
c
0nM
Period before=[23.5,24.5)
Period before=[24.5,25.5)
Period before=[25.5,26.5)
Period before=[26.5,27.5)
Period after=[23.5,24.5)
Period after=[24.5,25.5)
Period after=[25.5,26.5)
Period after=[26.5,27.5)
New Phase
Old Phase
.5 .75 0 .25 .5 .75 0 .25 .5

Supp. Fig. 4

Supplementary Figure 4. Circa-SCOPE is robust to period length variations

The data in Fig. 2d-e and Supplemenary Fig. 3a-c were binned according to cells' period length in 1 h intervals.

(a) Dexamethasone treated (100nM), binned by periods of the before fit.
(b) Dexamethasone treated (100nM), binned by periods of the after fit.
(c) Dexamethasone treated (1nM), binned by periods of the before fit.
(d) Dexamethasone treated (1nM), binned by periods of the after fit.
(e) Untreated, binned by periods of the before fit.
(f) Untreated, binned by periods of the after fit.
Source data are provided as Source Data file and in Supplementary Dataset 1.

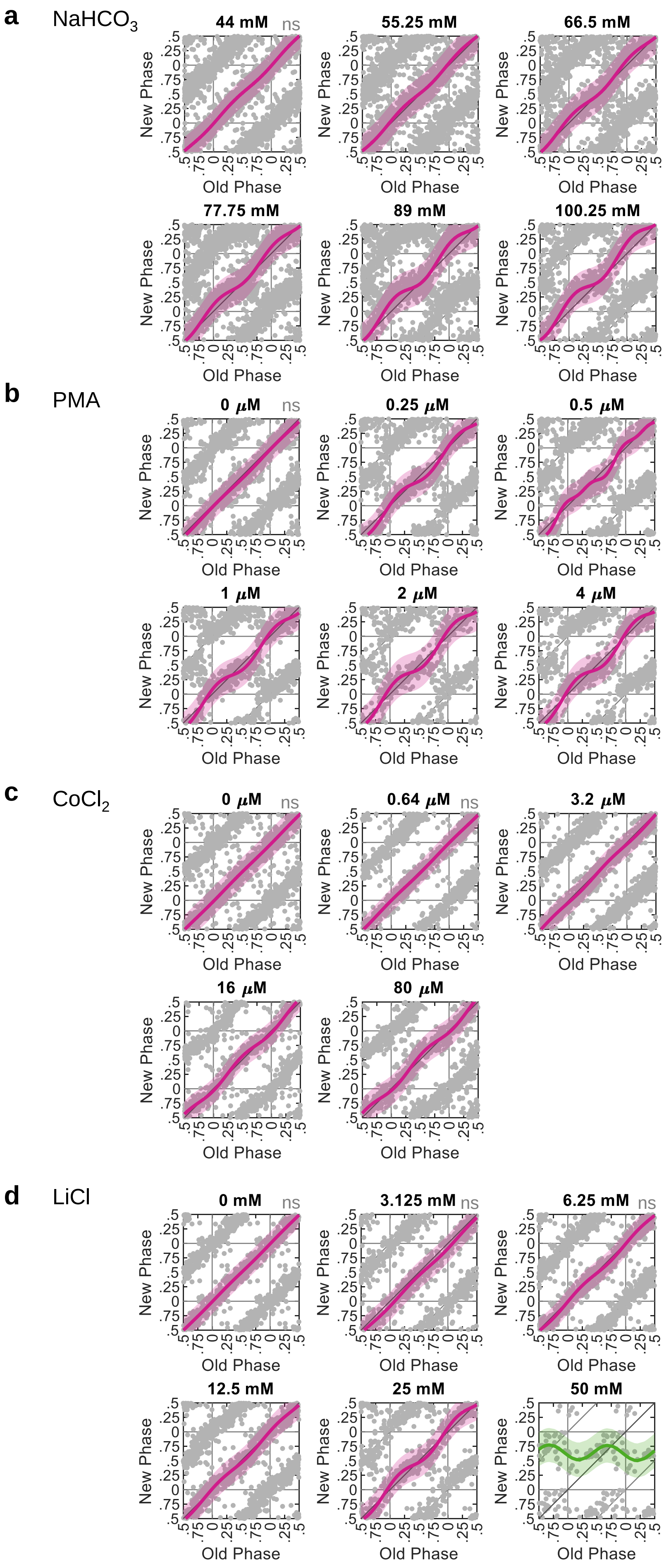
a
NaHCO3
44 mM
ns
55.25 mM
66.5 mM
77.75 mM
89 mM
100.25 mM
b
PMA
0 μM
ns
0.25 μM
0.5 μM
1 μM
2 μM
4 μM
c
CoCl2
0 μM
ns
0.64 μM
ns
3.2 μM
16 μM
80 μM
d
LiCl
0 mM
ns
3.125 mM
ns
6.25 mM
ns
12.5 mM
25 mM
50 mM
New Phase
Old Phase
.5
.25
0
.75
.5
.25
0
.75
.5

Supp. Fig. 5

Supplementary Figure 5. Circa-SCOPE can identify diverse PTC topologies
Cultures were treated with different concentrations of **(a)** $NaHCO_3$, **(b)** PMA, **(c)** LiCl, and **(d)** $CoCl_2$. Depicted are PTCs for each concentration. The PTCs of the relevant concentrations were replotted in Fig 3. (pink: type-1 resetting model ±95% confidence interval, green: type-0 resetting model ±95% confidence interval, ns: non-significant ($p>0.05$) in bootstrapping test for response, n=52-731 cells per condition).
Source data are provided in Supplementary Dataset 2.

Forskolin

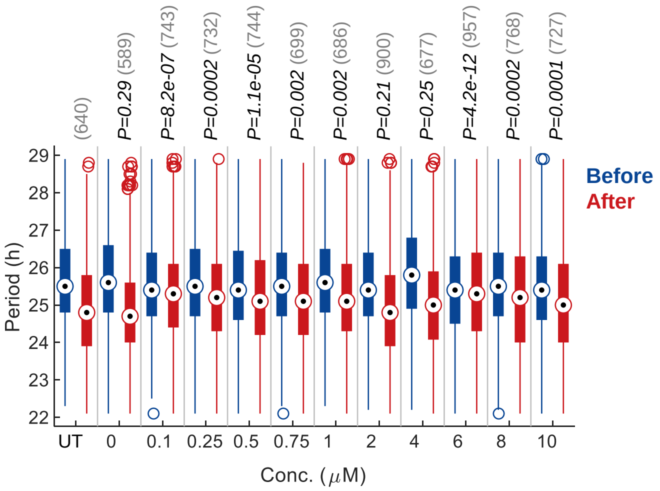

Supp. Fig. 6

Supplementary Figure 6. Period length comparison under different forskolin concentrations
Comparison of period length before and after treatment with different forskolin concentrations. (Two-sample two-sided Student's t-test between the period-change (After - Before) of the untreated (UT) control and the period-change in the treated group; n cells per condition indicated in brackets. Central circle: median; box: interquartile range (IQR); whiskers: extend to the maximal/minimal values within the range of ±1.5 IQR; circles: outliers).
Source data are provided in Supplementary Dataset 3.

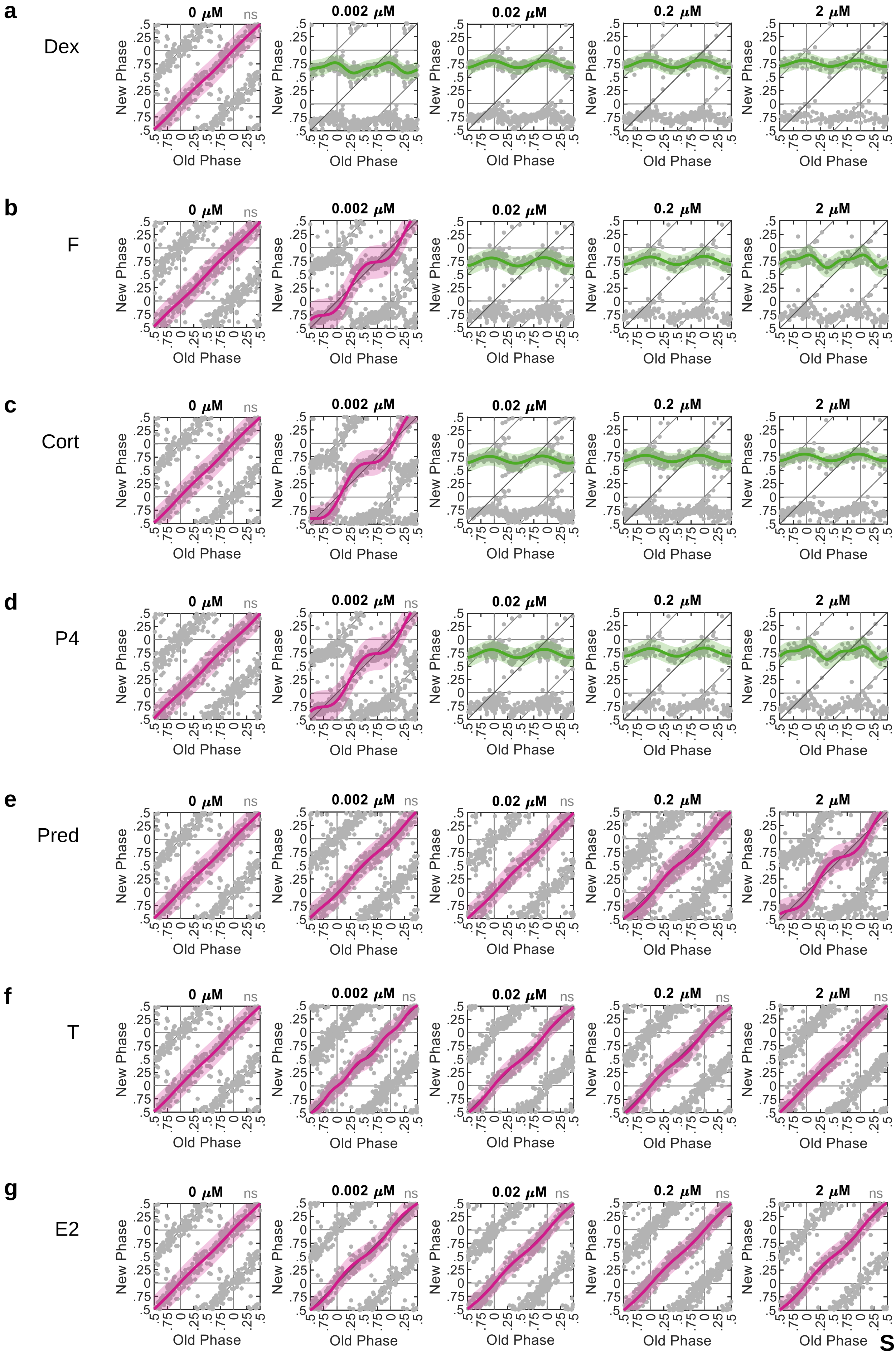

a
Dex
b
F
c
Cort
d
P4
e
Pred
f
T
g
E2
0 μM
0.002 μM
0.02 μM
0.2 μM
2 μM
ns
New Phase
Old Phase

Supp. Fig. 7

Supplementary Figure 7. Steroid drugs and hormones screen using Circa-SCOPE

Cultures were treated with 7 different steroids: **(a)** Dexamethasone (Dex), **(b)** Hydrocortisone (F), **(c)** Corticosterone (Cort), **(d)** Progesterone (P4), **(e)** Prednisone (Pred), **(f)** β-Estradiol (E2), and **(g)** Testosterone. Depicted are PTC for each concentration. Control (0 concentration) PTCs are identical for all compounds and were performed with DMSO, except hydrocortisone which was diluted in ethanol and hence have a different control. (Pink: type-1 resetting model ±95% confidence interval, green: type-0 resetting model ±95% confidence interval, ns: non-significant ($p>0.05$) in bootstrapping test for response, n=83-346 cells per condition).

Source data are provided in Supplementary Dataset 4.

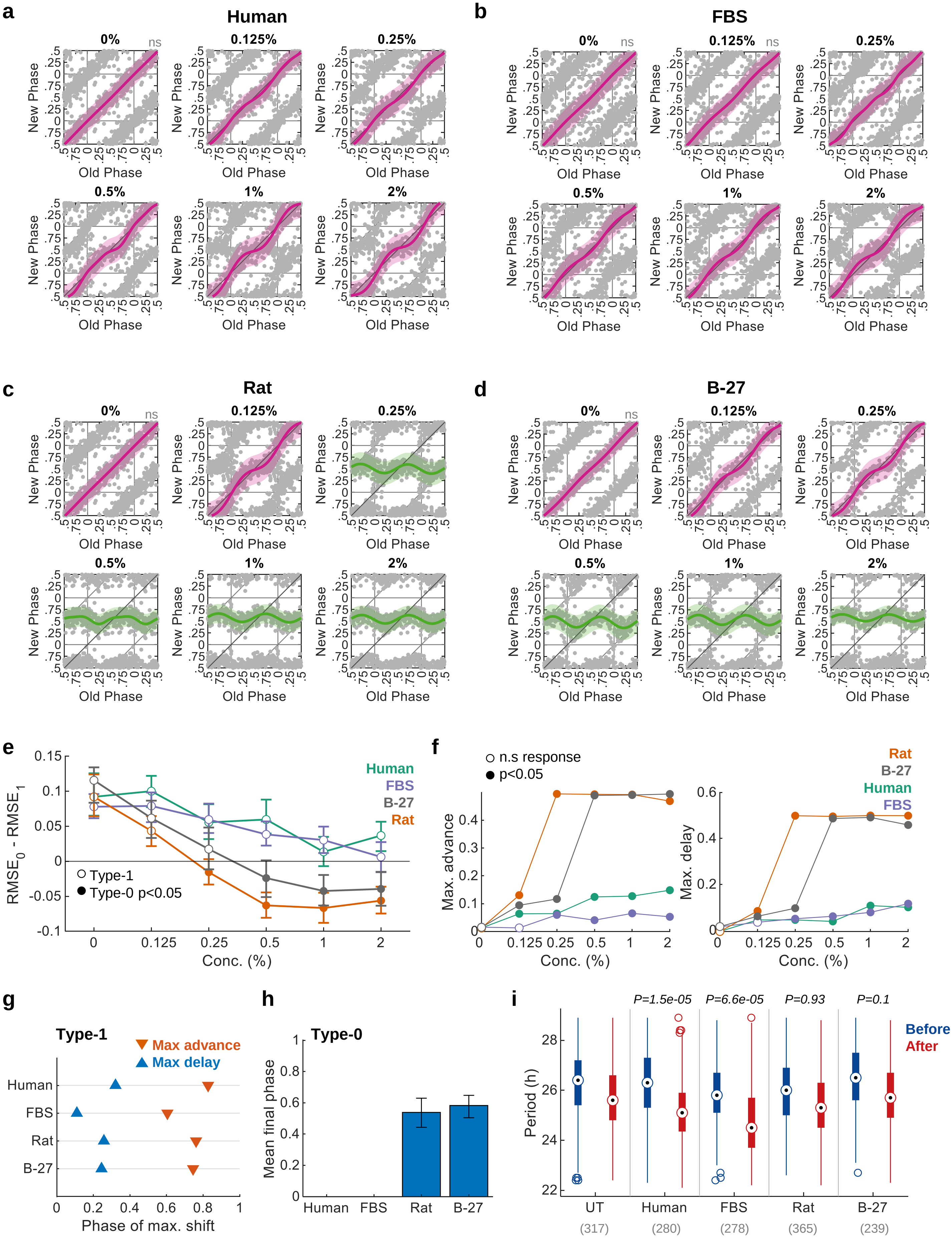

a
Human
b
FBS
c
Rat
d
B-27
0%
0.125%
0.25%
0.5%
1%
2%
ns
New Phase
Old Phase
e
Human
FBS
B-27
Rat
Type-1
Type-0 p<0.05
$RMSE_0 - RMSE_1$
Conc. (%)
f
n.s response
p<0.05
Rat
B-27
Human
FBS
Max. advance
Max. delay
Conc. (%)
g
Type-1
Max advance
Max delay
Human
FBS
Rat
B-27
Phase of max. shift
h
Type-0
Mean final phase
Human
FBS
Rat
B-27
i
P=1.5e-05
P=6.6e-05
P=0.93
P=0.1
Before
After
Period (h)
UT
Human
FBS
Rat
B-27
(317)
(280)
(278)
(365)
(239)

Supp. Fig. 8

Supplementary Figure 8. PTC analysis of different serums

(a-d) Cultures were treated with different concentrations of **(a)** Human serum, **(b)** Fetal Bovine Serum (FBS), **(c)** Rat serum, and **(d)** B-27 supplement. Depicted are PTCs for each concentration. The PTCs for 2% were replotted in Fig 5. (Pink: type-1 resetting model ±95% confidence interval, green: type-0 resetting model ±95% confidence interval, ns: non-significant ($p>0.05$) in bootstrapping test for response, n=193-474cells per condition).

(e) Model selection for each serum across the tested concentrations. Dots represent the observed $RMSE_0$-$RMSE_1$, error bars represent the 95% confidence interval from bootstrapping test. Filled dots stand for significant type-0 fit according to bootstrapping test.

(f) Maximal phase advance (left) and maximal phase delay (right) for each serum across the tested concentrations. Empty dots represent non-significant phase shift based on bootstrapping test.

(g) Phase of maximal shift for the maximal concentration with significant type-1 resetting with each serum.

(h) Mean final phase for the maximal concentration with significant type-0 resetting with each serum (Error bars represent the range of final phases from the fit). Data for treatments without significant type-0 response in any concentration are omitted.

(i) Comparison of period length before and after treatment with 2% of each serum. (Two-sample two-sided Student's t-test between the period-change (After - Before) of the untreated (UT) control and the period-change in the treated group; n cells per condition indicated in brackets. Central circle: median; box: interquartile range (IQR); whiskers: extend to the maximal/minimal values within the range of ±1.5 IQR; circles: outliers).

Source data are provided as Source Data file and in Supplementary Dataset 5.

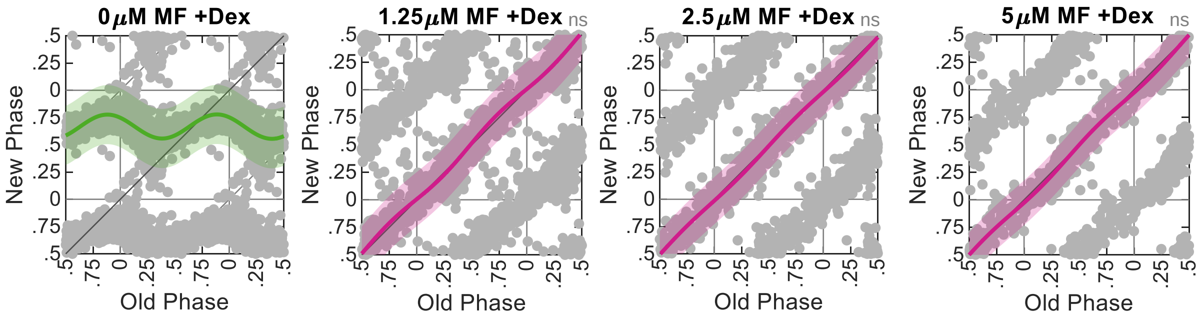
0μM MF +Dex
1.25μM MF +Dex
ns
2.5μM MF +Dex
ns
5μM MF +Dex
ns
New Phase
Old Phase
.5
.25
0
.75

Supp. Fig. 9

Supplementary Figure 9. Glucocorticoid and progesterone receptor inhibition abolishes dexamethasone-induced clock resetting

PTCs of cultures in response to 1nM dexamethasone (Dex), with or without pretreatment of different mifepristone (MF) concentrations (in µM). (Pink: type-1 resetting model ±95% confidence interval, green: type-0 resetting model ±95% confidence interval, ns: not significant ($p>0.05$) in bootstrapping test for response, n=288-532 cells per condition).

Source data are provided in Supplementary Dataset 7.

Supplementary Tables

Supplementary Table 1: Summary of resetting experiments results

Summary of the resetting parameters retrieved for different compounds using CircaSCOPE in this study. All phase data are given relative to the period length.

Compound	Period effect	Type-1 to 0 transition	Phase of max. delay (type-1)	Phase of max. advance (type-1)	Mean final phase (type-0)
**Steroids:**					
Dexamethasone	Decrease	1-2nM	-	-	0.78
Prednisone	~	>2uM	0.44	0.94	-
Corticosterone	Decrease	0.002-0.02uM	0.39	0.89	0.73
Cortisol	Decrease	0.002-0.02uM	0.49	0.99	0.78
Progesterone	Increase	0.02-0.2uM	0.37	0.87	0.72
β-Estradiol	Increase	-	-	-	-
Testosterone	Increase	-	-	-	-
**Serum:**					
Human	Decrease	>2%	0.30	0.80	-
Rat	Decrease	0.125-0.25%	0.28	0.78	0.56
FBS	~	>2%	0.08	0.69	-
B-27	~	0.25-0.5%	0.19	0.82	0.57
**Miscellaneous:**					
$NaHCO_3$	Increase	≳100.25mM*	0.09	0.62	-
PMA	~	≳4uM	0.04	0.71	-
LiCl	Increase	25-50mM	0.16	0.67	0.68
$CoCl_2$	Decrease	>80uM	0.52	0.02	-
Forskolin	~	2-4uM	0.39	0.90	0.70

*on background of 44mM (i.e., 56.75 increase)

Supplementary Table 2: List of reagents used for resetting experiments

Material	Catalog number	Manufacturer	Solvent
**$CoCl_2$**	232696	Sigma	Water
**Corticosterone**	C2505	Sigma	DMSO
**Dexamethasone**	D4902	Sigma	DMSO
**FBS**	12657-029	Gibco	-
**Forskolin**	BML-CN100-0010	Enzo Life Sciences	DMSO
**Human serum**	H4522	Sigma	-
**Hydrocortisone**	H0888	Sigma	Ethanol
**LiCl**	ICN19401090	MP Biomedicals	Water
**Mifepristone**	459980010	ACROS ORGANICS	DMSO
**$NaHCO_3$ (7.5% solution)**	S8761	Sigma	-
**Phorbol-12-myristate-13-acetate**	356150010	ACROS ORGANICS	DMSO
**Prednisone**	448950050	ACROS ORGANICS	DMSO
**Progesterone**	P0130	Sigma	DMSO
**Testosterone**	86500	Sigma	DMSO
**β-Estradiol**	E8875	Sigma	DMSO
